# COVID-19, uncertainty, and moral experiments

**DOI:** 10.1007/s40656-020-00360-9

**Published:** 2021-01-13

**Authors:** Michael Klenk, Ibo Van de Poel

**Affiliations:** Delft University of Technology, Jafaalaan 5, 2628 BX Delft, The Netherlands

**Keywords:** Moral dilemmas, Uncertainty, Rationality

## Abstract

Pandemics like COVID-19 confront us with decisions about life and death that come with great uncertainty, factual as well as moral. How should policy makers deal with such uncertainty? We suggest that rather than to deliberate until they have found the right course of action, they better do moral experiments that generate relevant experiences to enable more reliable moral evaluations and rational decisions.

## Introduction

The COVID-19 pandemic confronts societies around the globe with daunting decisions about life and death. In order to *save lives* (and prevent an overload of the healthcare system), governments had to resort to indiscriminate mitigation strategies like population-wide lockdown. However, those countermeasures threaten the goal of *saving livelihoods* because, amongst other things, they put an extraordinary economic and psychological strain on some people. Therefore, deciding about how to deal with COVID-19 seems dilemmatic: incomparable values like the value of life and the value of freedom are at stake, and they cannot be satisfied at the same time.

This decision dilemma creates great anxiety and uncertainty, for virtually all members of affected societies It creates not only factual uncertainty about the future and the consequences of our actions but also moral uncertainty, about what is best. In what follows, we will focus on the decision situation faced by policymakers and invite the reader to assume the perspective of someone who has to take a decision on how to combate the pandemic in light of that uncertainty, perhaps as a custodian and representative for other parts of society. Given the stakes, this moral uncertainty also has an existential dimension: decision makers must feel that it matters utmost what they choose; at the same time, they are or at least should be greatly uncertain what to do. It might even seem that whatever they do, they make a (morally) wrong choice. The traditional (philosophical) response to such dilemmas would seem to either think deeper, and deliberate more about what is best, or to conclude that there is no rationally best course of action, so that any choice is just an uncertain jump into the future.^1^

In this short paper, we tackle the question of whether there could be a superior alternative. We wish to invite the reader to consider whether what we call moral experimentation could be an acceptable and helpful approach for policy makers to dealing with the moral uncertainties of a pandemic. To indicate why moral experimentation could be an acceptable alternative, we first need to unravel the moral uncertainty that currently confronts us.

## Moral uncertainty

We suggest that we take the anxieties and uncertainties created by a pandemic seriously. They point to a real problem with making reliable moral judgements in situations of moral uncertainty. In short, we *need* relevant experiences to deal with pandemics, but we *lack* them. That creates a problem with moral uncertainty.

Recent insights from moral philosophical and moral psychological suggest that relevant moral experiences are helpful, if not required, for reliable moral judgements. For example, scholars in experimental moral philosophy generally acknowledge indirectly that the reliability of moral judgements is enhanced by a realistic stimulus when they criticise all too neatly described moral thought experiments as elicitors of moral judgements (Pölzler 2018). Anecdotal evidence from outside the lab comes from cases in which lively first-hand experiences drastically change the moral judgements of individuals. We are not aware of examples where people changed their moral views in the context of a disease, but the general case of moral conversion after direct experience seems common, for example, when people make a commitment to vegetarianism after witnessing a slaughter. Moral judgements are also increasingly understood as extended in time or reflective in moral psychology, so that a post hoc evaluation of several experiences constitutes a moral judgement (Sauer 2017). The importance of first-hand experience in making moral judgements is also stressed from a different angle in debates about moral testimony. Scholars of moral testimony suggest that moral testimony cannot confer justification in place of first-hand experience of the matter, even if it comes from reliable sources (Hills 2009). A fairly general theory that (in parts) supports our claim about the importance of first-hand moral experience is standpoint theory. Standpoint theorists argue that there are particular experientially advantaged perspectives in evaluating and describing the world (Fricker 2013). The pertinent point for our purposes is that an individual’s standpoint is autoritative on some topic (if it is) in virtue of the individual’s particular and idiosyncratic experiences. This suggests that there are particularly authoritative standpoints on issues to do with pandemics: those that were arrived at by experiencing a pandemic before (probably especially those who experienced a relevantly similar pandemic). Moral education researchers have noted the central and elucidating role played by engaging narratives that intertwine with people’s experiences in teaching moral precepts (Tappan and Brown 1989). These claims underscore the idea that experiences with a situation are required for reliable moral judgement.

The problem with COVID-19 and other pandemics, is that decision makerslack the required experiences needed to make reliable evaluations. Decision-makers today have never before had to decide about population-wide lockdowns and, at least in parts of the Western world, they did not have to confront the lives vs livelihood tradeoff in such magnitude, and such vividness. The heated debate about suitable COVID-19 countermeasures might be interpreted to be—at least in part—a reflection of our collective inexperience and thus uncertainty about the proper moral evaluation of the crisis and our countermeasures.

## Moral experiments

Collectively, we may wish to develop and systematize a methodology that we call ‘moral experiments’ to improve our decisions in times of crisis. We will now suggest why this will help us systematically generate relevant moral experiences.

Moral experiments are structured and goal-directed processes through which participants adduce moral experiences that inform their moral decisions. They are reminiscent of Mill’s experiments in living (Mill 1869), but in contrast to Mill’s individual endeavours, moral experiments are collective and structured. They also differ from ‘merely’ or unsystematically gaining new experiences in several respects. First of all, moral experiments are based on a hypothesis that is tested through the experiment. Therefore, rational experimentation is not just trial-and-error but guided by intelligence (Dewey 1938). Moreover, proper moral experimentation is aimed at moral learning, which means that arrangements need to be put in place so that the new experiences gained are gathered, discussed and interpreted in order to facilitate moral learning (van de Poel 2017). We can sketch the core features of a moral experiment as follows:

A moral experiment is a (1) collective, (2) structured, and (3) goal-directed process aimed at enabling different morally relevant direct experiences of a particular moral problem in two or more otherwise similar groups of subject to improve moral understanding about that moral problem with (4) an institution in place to gather, discuss, and interpret the findings of that process.^2^

The variation in COVID-19 countermeasures around the globe today appear similar to moral experiments already. However, this lacks the systematic approach that is integral to apt moral experimentation. By systematically pitting seemingly incomparable outcomes against one another in comparable situations, decision-makers could gain relevant moral experience fast and more reliably. For example, people’s considered judgements about stringent lockdowns (e.g. in Italy) vs lax regimes that cost more lives (e.g. in the US) can be used to update the moral evaluation of each respective outcome. In this way, the seeming incomparability of both options might possibly vanish after experimentation.

Comparisons between countries are currently, however, difficult. Sweden has, for example, chosen a somewhat more lax approach than other countries stressing individual responsibility rather than strict government measures. The success of this strategy is controversial (e.g. Lindström 2020). The reported death rate per million inhabitants is currently (December 2020) lower than countries like the US, Spain and Italy, but much higher than in neighboring countries like Norway and Denmark, and well above the world average.^3^ Public support for government measures is around the median of 14 advanced economies, higher than in the UK and US, but lower than in Germany and Denmark.^4^ However, without a systematic set-up of such national ‘experiments’, outcomes remain hard to compare, also due to geographical, demographic and cultural differences.

More systematic moral experimentation would first of all require recognizing the experimental nature of the current responses to COVID-19, so that experiences are systematically used as input for moral deliberation and learning. It would also require setting up experiments in a way that outcomes can be more easily and reliably compared. One can, for example, imagine that countries decide to experiment with different responses in different parts of a country in order to ensure comparability of outcomes and experiences.

## Conclusion

Pandemics like COVID-19 confront us with decisions about life and death that involve great moral uncertainty. This moral uncertainty may be so overwhelming that we either freeze (and fail to act) or that we engage in painstaking, but inconclusive, deliberations. We suggested that it might be better to embrace moral uncertainty in such situations and to engage in moral experimentation.

Naturally, a responsible use of moral experimentation requires settling many more questions that we are aiming to address in future work. For example, there are questions about how best to adopt the method to national or local circumstances. For now, we hope for this note to be a stimulus for further deliberation about whether moral experimentation can help us gain over time new factual insights as well as the moral experiences that are needed for better and more reliable moral judgements in times of crisis.

## Footnotes

1 Philosophical discussion on moral dilemmas tend to focus on whether such dilemma really exist or not. Normative decision theory may offer guidance in how to deal with moral uncertainty, but in order to compute a choice’s expected value, we must first morally evaluate its possible outcome. However, our evaluations are not (at least not initially) reliable in situations like the COVID-19 pandemic, where we have only a little experience – and so we cannot apply normative decision theory just yet, as we will show below.

2 The fact that moral experiments require direct experience of a moral problem, that they collective, and connected with an institution aimed at incorporating the insights gained from the experiment into practical decision making about the moral problem at hand is what distinguishes moral experiments from familiar philosophical thought experiments about morality.

3 Worldometer (https://www.worldometers.info/coronavirus/) reports the following numbers of deaths per million population on 14 December 2020: Sweden 742; US 923; UK 943; Italy 1068; Spain 1018; Germany 267; Denmark 162; Norway 71; reported world average is 207.9. Note however that reporting and criteria may be different between countries, so that numbers ae hard to compare.

4 https://www.pewresearch.org/global/2020/08/27/most-approve-of-national-response-to-covid-19-in-14-advanced-economies/Accessed 14 December 2020.
